# Supplementary material for: Natural History of Aerosol-Induced Ebola Virus Disease in Rhesus Macaques
Source: Viruses. 2021 Nov 17;13(11):2297. doi: 10.3390/v13112297 (PMC8619410; doi:10.3390/v13112297)
Supplement: Supplementary file 1 [file viruses-13-02297-s001.zip › viruses-1429301-supplementary.pdf]

Table S1. Characteristics of rhesus macaques in the natural history study of EVD. Initial body weight, age, gender, day of death and euthanasia determination for each animal in natural history study.

Characteristic	NHP1	NHP2	NHP3	NHP4
Body weight (kg)	5.8	6.5	5.5	9.4
Age (years)	4	5	4	4
Gender	Female	Male	Female	Male
NHP name in ref. [34]	NH3F	NH1M	NH4F	NH2M
AGI Conc. (PFU/ml)	497	316	691	313
Minute vol. (ml/min)	1060	1410	1385	1909
Delivery Dose (PFU)	878	743	1595	996
Day of death	DPE 8	DPE 7	DPE 10	DPE 7
Euthanization	Yes	No	Yes	No

Table S2. Clinical observations of rhesus macaques during the natural history study of EVD. Assessment scores are normal (0), very mild (1), mild (2), moderate (3), and severe (4) for responsiveness/behavior and seizures. Assessment scores are normal (0), mild (1), moderate (2), and severe (3) for biscuit consumption, fruit consumption, condition of stool, edema, rash, tremors, posture, respiration, and bleeding. Urine output and nasal exudate are marked yes (Y) or no (N). Neurological dysfunction is marked by either none, ocular, auditory, motor, or all. Determination of disease stage includes mean clinical score of all animals and additional analysis. Subclinical stage (DPE 0-4) is defined with mean clinical score of zero until fever is detected. The clinical stage of EVD progression (DPE 5-6) involves detection of primarily fever, and further characterized by viremia and alkalosis with blood pH > 7.5. Post DPE 6, the decompensated stage is marked by mean clinical score ≥2.0 as well as high levels of D-dimers, cytokines, liver and kidney functions markers.

	GROUPS	DPE -3	DPE -2	DPE -1	DPE 0	DPE 1	DPE 2	DPE 3	DPE 4	DPE 5. AM	DPE 5. PM	DPE 6. AM	DPE 6. PM	DPE 8. AM	DPE 8. PM	DPE 9. AM	DPE 9. PM	DPE 10. AM	DPE 10. PM
STAGE OF DISEASE	ALL ANIMALS	NO EXPOSURE	NO EXPOSURE	NO EXPOSURE	SUBCLIN	SUBCLIN	SUBCLIN	SUBCLIN	SUBCLIN	CLINICAL	CLINICAL	CLINICAL	CLINICAL	DECOMP	DECOMP	DECOMP	DECOMP	DECOMP	DECOMP
Biscuit Consumption	ALL ANIMALS	0.0	0.0	0.0	0.0	0.0	0.0	0.0	0.0	0.0	0.0	0.8	2.8	3.0	3.0	N/A	3.0	3.0	3.0
	SD	0.0	0.0	0.0	0.0	0.0	0.0	0.0	0.0	0.0	0.0	1.5	0.5	0.0	0.0	N/A	N/A	N/A	N/A
Fruit Consumption	ALL ANIMALS	0.0	0.0	0.0	0.0	0.0	0.0	0.0	0.0	0.0	0.5	0.0	1.3	3.0	3.0	2.0	3.0	3.0	3.0
	SD	0.0	0.0	0.0	0.0	0.0	0.0	0.0	0.0	0.0	1.0	0.0	1.5	0.0	0.0	N/A	N/A	N/A	N/A
Condition of Stool	ALL ANIMALS	0.0	0.0	0.0	0.0	0.0	0.0	0.0	0.0	0.0	0.0	0.0	1.5	3.0	3.0	N/A	3.0	3.0	3.0
	SD	0.0	0.0	0.0	0.0	0.0	0.0	0.0	0.0	0.0	0.0	0.0	1.7	0.0	0.0	N/A	N/A	N/A	N/A
Urine Output	ALL ANIMALS	Y	Y	Y	Y	Y	Y	Y	Y	Y	Y	Y	Y	Y/N	Y/N	Y	Y	Y	Y
	SD	N/A	N/A	N/A	N/A	N/A	N/A	N/A	N/A	N/A	N/A	N/A	N/A	N/A	N/A	N/A	N/A	N/A	N/A
Nasal Exudate	ALL ANIMALS	N	N	N	N	N	N	N	N	N	N	N	N	N	N	N	N	N	N
	SD	N/A	N/A	N/A	N/A	N/A	N/A	N/A	N/A	N/A	N/A	N/A	N/A	N/A	N/A	N/A	N/A	N/A	N/A
Edema	ALL ANIMALS	0.0	0.0	0.0	0.0	0.0	0.0	0.0	0.0	0.0	0.0	0.0	0.0	1.0	0.0	0.0	0.0	0.0	0.0
	SD	0.0	0.0	0.0	0.0	0.0	0.0	0.0	0.0	0.0	0.0	0.0	0.0	1.4	N/A	N/A	N/A	N/A	N/A
Rash	ALL ANIMALS	0.0	0.0	0.0	0.0	0.0	0.0	0.0	0.0	0.0	0.0	0.0	0.5	2.5	3.0	3.0	3.0	3.0	3.0
	SD	0.0	0.0	0.0	0.0	0.0	0.0	0.0	0.0	0.0	0.0	0.0	1.0	0.7	N/A	N/A	N/A	N/A	N/A
Tremors	ALL ANIMALS	0.0	0.0	0.0	0.0	0.0	0.0	0.0	0.0	0.0	0.0	0.0	0.0	0.0	0.0	0.0	0.0	0.0	1.0
	SD	0.0	0.0	0.0	0.0	0.0	0.0	0.0	0.0	0.0	0.0	0.0	0.0	0.0	N/A	N/A	N/A	N/A	N/A
Neurological Dysfunction	ALL ANIMALS	None	None	None	None	None	None	None	None	None	None	None	None	Motor/None	ALL	ALL	ALL	ALL	ALL
	SD	N/A	N/A	N/A	N/A	N/A	N/A	N/A	N/A	N/A	N/A	N/A	N/A	N/A	N/A	N/A	N/A	N/A	N/A
Responsiveness/Behavior	ALL ANIMALS	0.0	0.0	0.0	0.0	0.0	0.0	0.0	0.0	0.0	0.0	0.0	0.5	1.0	2.5	1.0	2.0	3.0	4.0
	SD	0.0	0.0	0.0	0.0	0.0	0.0	0.0	0.0	0.0	0.0	0.0	0.6	1.4	2.1	N/A	N/A	N/A	N/A
Posture	ALL ANIMALS	0.0	0.0	0.0	0.0	0.0	0.0	0.0	0.0	0.0	0.0	0.0	0.0	0.5	1.5	1.0	3.0	3.0	3.0
	SD	0.0	0.0	0.0	0.0	0.0	0.0	0.0	0.0	0.0	0.0	0.0	0.0	0.7	2.1	N/A	N/A	N/A	N/A
Respiration	ALL ANIMALS	0.0	0.0	0.0	0.0	0.0	0.0	0.0	0.0	0.0	0.0	0.0	0.0	0.5	1.5	0.0	0.0	0.0	1.0
	SD	0.0	0.0	0.0	0.0	0.0	0.0	0.0	0.0	0.0	0.0	0.0	0.0	0.7	2.1	N/A	N/A	N/A	N/A
Bleeding	ALL ANIMALS	0.0	0.0	0.0	0.0	0.0	0.0	0.0	0.0	0.0	0.0	0.0	0.0	0.0	0.0	0.0	0.0	0.0	0.0
	SD	0.0	0.0	0.0	0.0	0.0	0.0	0.0	0.0	0.0	0.0	0.0	0.0	0.0	0.0	N/A	N/A	N/A	N/A
Seizures	ALL ANIMALS	0.0	0.0	0.0	0.0	0.0	0.0	0.0	0.0	0.0	0.0	0.0	0.0	0.0	0.0	0.0	0.0	0.0	0.0
	SD	0.0	0.0	0.0	0.0	0.0	0.0	0.0	0.0	0.0	0.0	0.0	0.0	0.0	0.0	N/A	N/A	N/A	N/A
MEAN CLINICAL SCORES	ALL ANIMALS	0.0	0.0	0.0	0.0	0.0	0.0	0.0	0.0	0.0	0.0	0.0	0.5	2.0	5.5	2.0	5.0	6.0	8.0
	SD	0.0	0.0	0.0	0.0	0.0	0.0	0.0	0.0	0.0	0.0	0.0	0.6	2.8	6.4	N/A	N/A	N/A	N/A

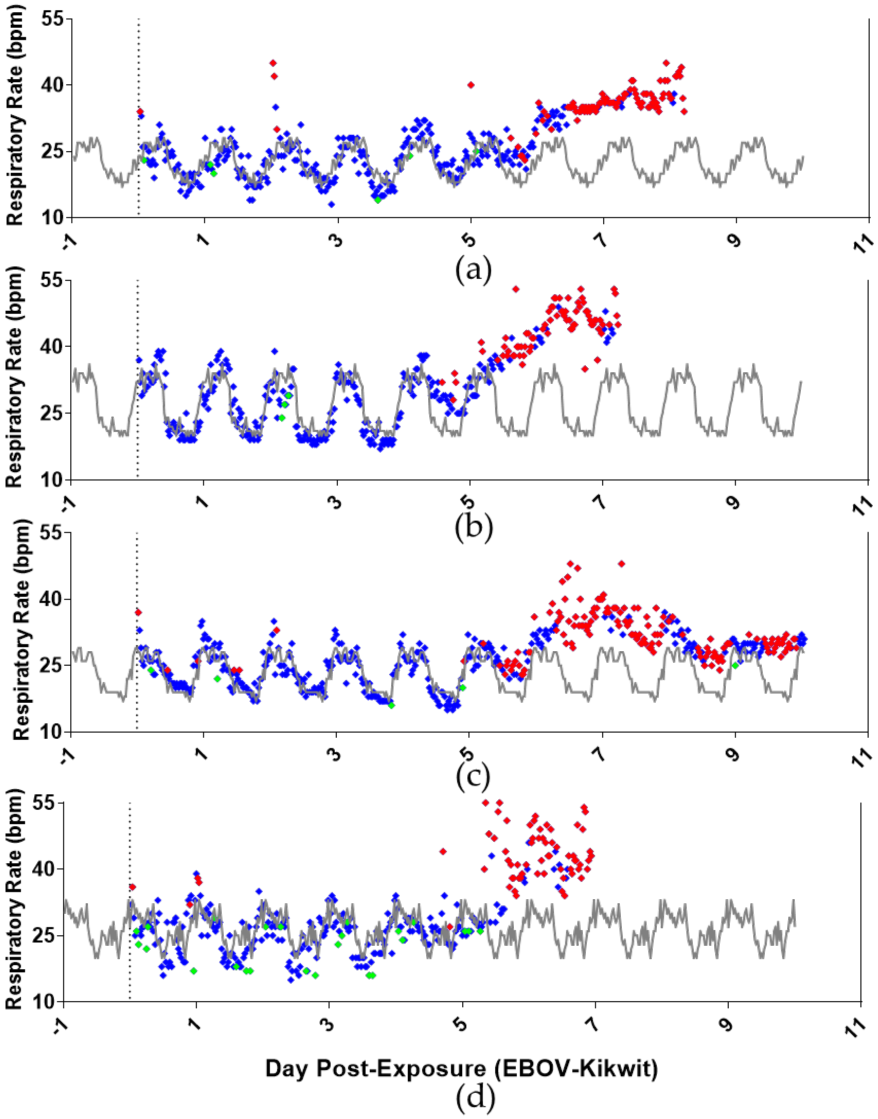

(d)

Figure S1. Telemetric respiratory rate detection of aerosol EBOV exposed rhesus macaques (a) NHP1 (b) NHP2 (c) NHP3 (d) NHP4. Vertical grey dotted line marks DPE 0, — BL (Baseline), ♦ HS (Values significantly higher: >3.0 SD from corresponding baseline) for longer than 2h, ♦ LS (Values significantly lower: < 3.0 SD from corresponding baseline) for longer than 2h, ♦ Respiratory rate for longer than 2h.

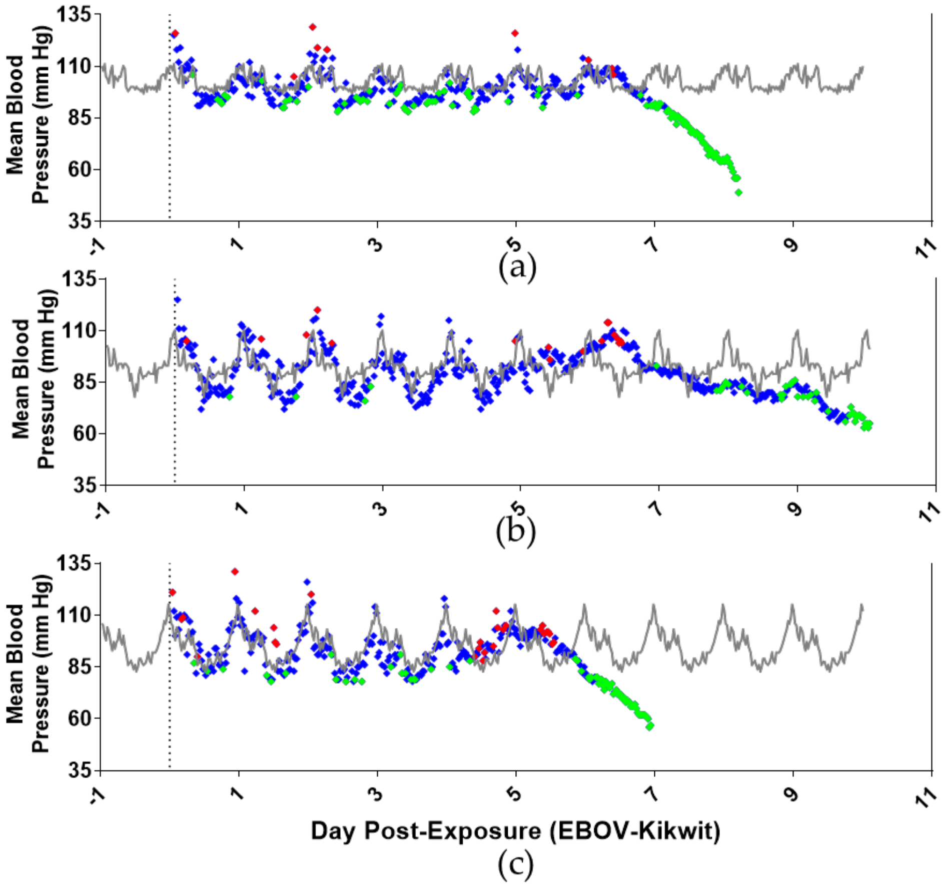

Figure S2. Telemetric detection of mean blood pressure of aerosol EBOV exposed rhesus macaques (a) NHP1 (b) NHP3 (c) NHP4. NHP2 had a nonfunctional pressure sensor, so no mean blood pressure is available. Vertical grey dotted line marks DPE 0, — BL (Baseline), ♦ HS (Values significantly higher: >3.0 SD from corresponding baseline) for longer than 2h, ♦ LS (Values significantly lower: < 3.0 SD from corresponding baseline) for longer than 2h, ♦ Blood pressure for longer than 2h.

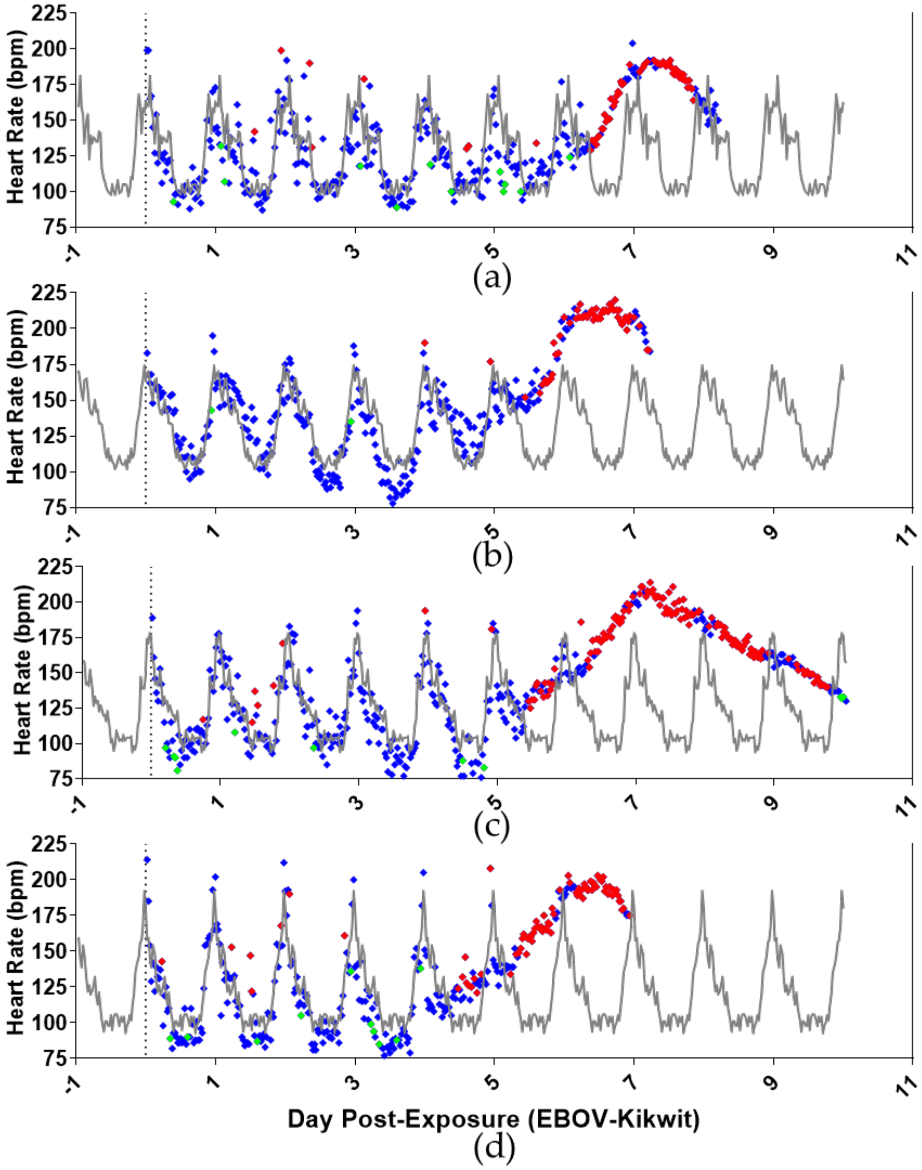

Figure S3. Telemetric heart rate detection of aerosol EBOV exposed rhesus macaques (a) NHP1 (b) NHP2 (c) NHP3 (d) NHP4. Vertical grey dotted line marks DPE 0, — BL (Baseline), ♦ HS (Values significantly higher: >3.0 SD from corresponding baseline) for longer than 2h, ♦ LS (Values significantly lower: < 3.0 SD from corresponding baseline) for longer than 2h, ♦ Heart rate for longer than 2h.

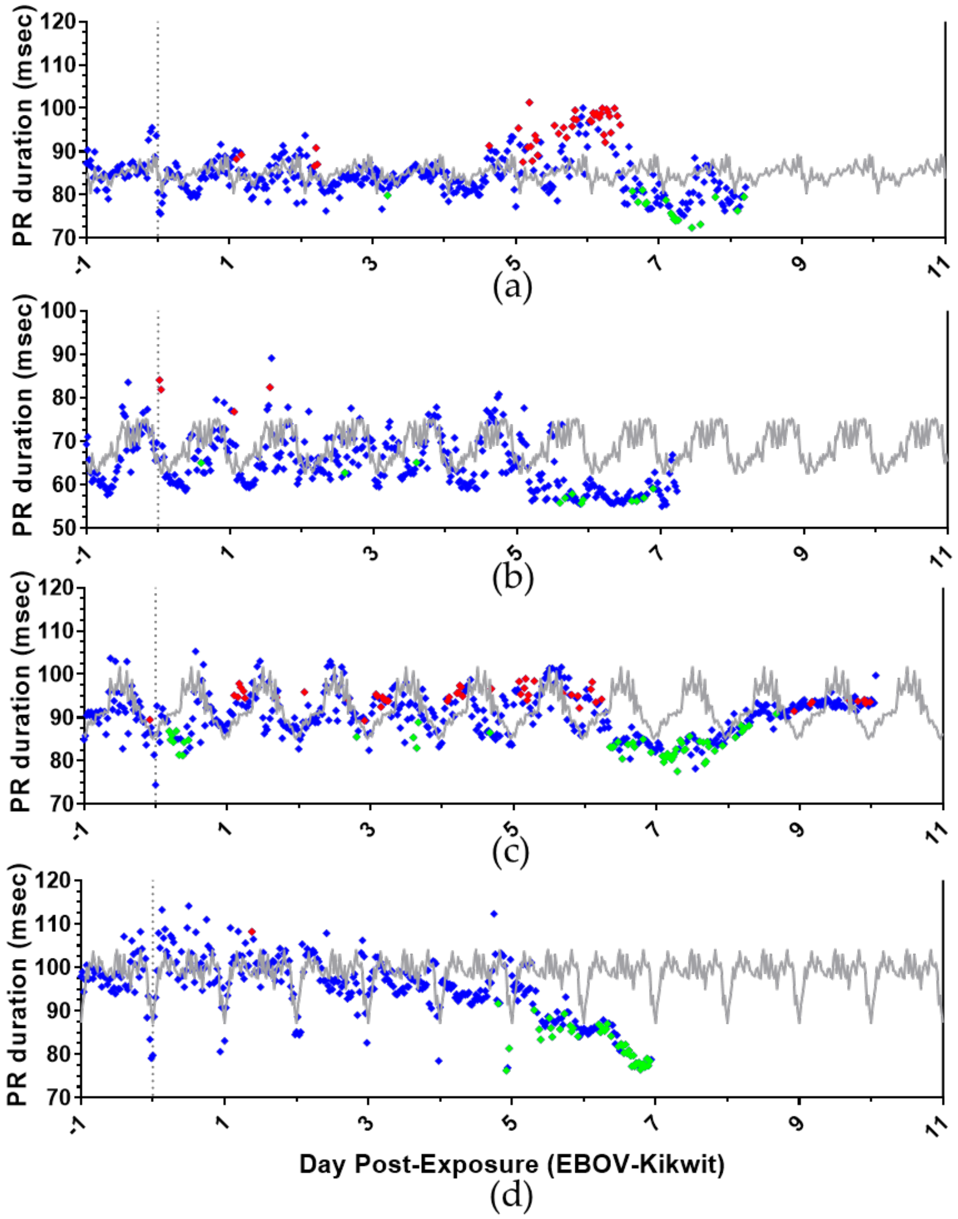

Figure S4. Telemetric PR duration detection of aerosol EBOV exposed rhesus macaques (a) NHP1 (b) NHP2 (c) NHP3 (d) NHP4. Vertical grey dotted line marks DPE 0, — BL (Baseline), ♦ HS (Values significantly higher: >3.0 SD from corresponding baseline), ♦ LS (Values significantly lower: < 3.0 SD from corresponding baseline), ♦ (Values within 3.0 SD from corresponding baseline) for PR duration measured in milliseconds.

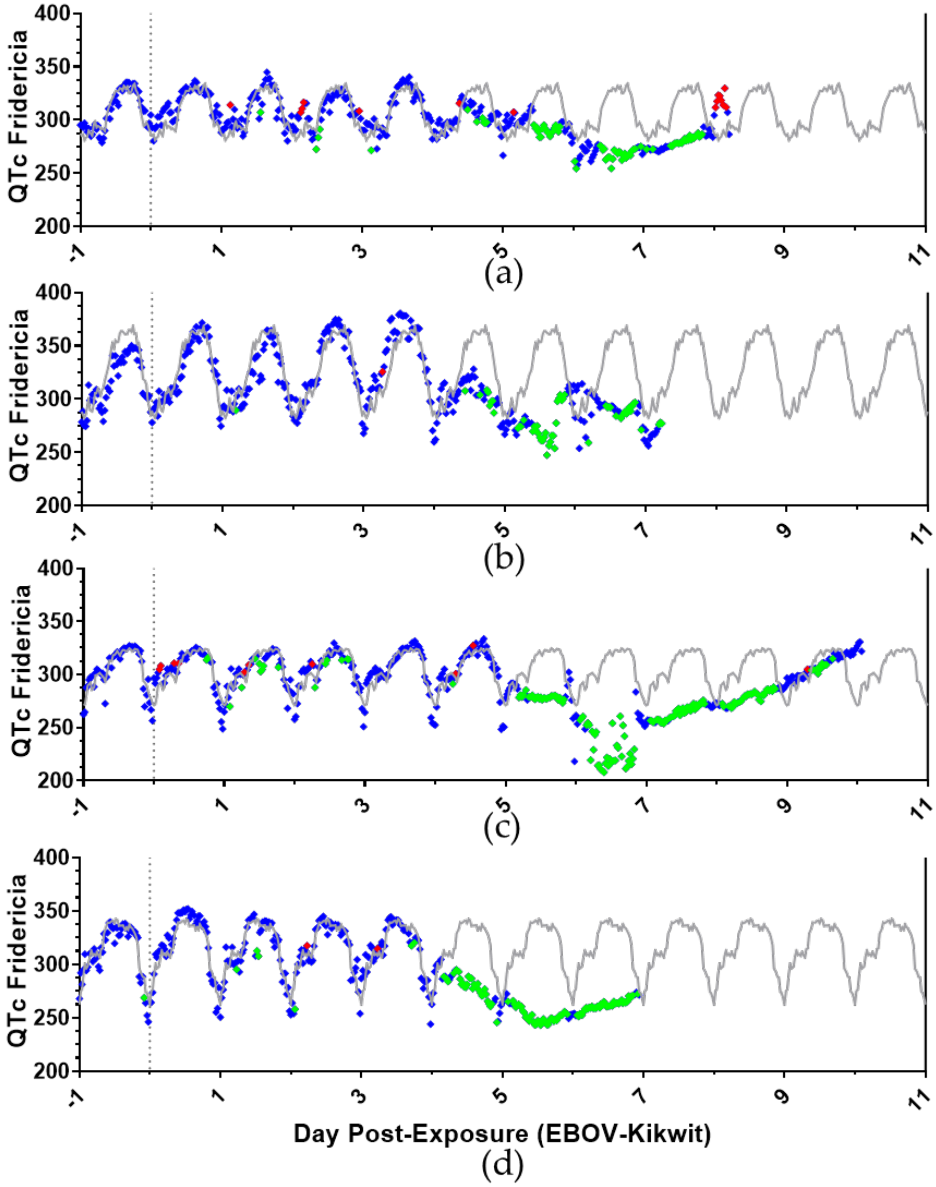

Figure S5. Telemetric QT corrected Fridericia duration detection of aerosol EBOV exposed rhesus macaques (a) NHP1 (b) NHP2 (c) NHP3 (d) NHP4. Vertical grey dotted line marks DPE 0, — BL (Baseline), ♦ HS (Values significantly higher: >3.0 SD from corresponding baseline), ♦ LS (Values significantly lower: < 3.0 SD from corresponding baseline), ♦ (Values within 3.0 SD from corresponding baseline) for QT corrected Fridericia duration measured in milliseconds.

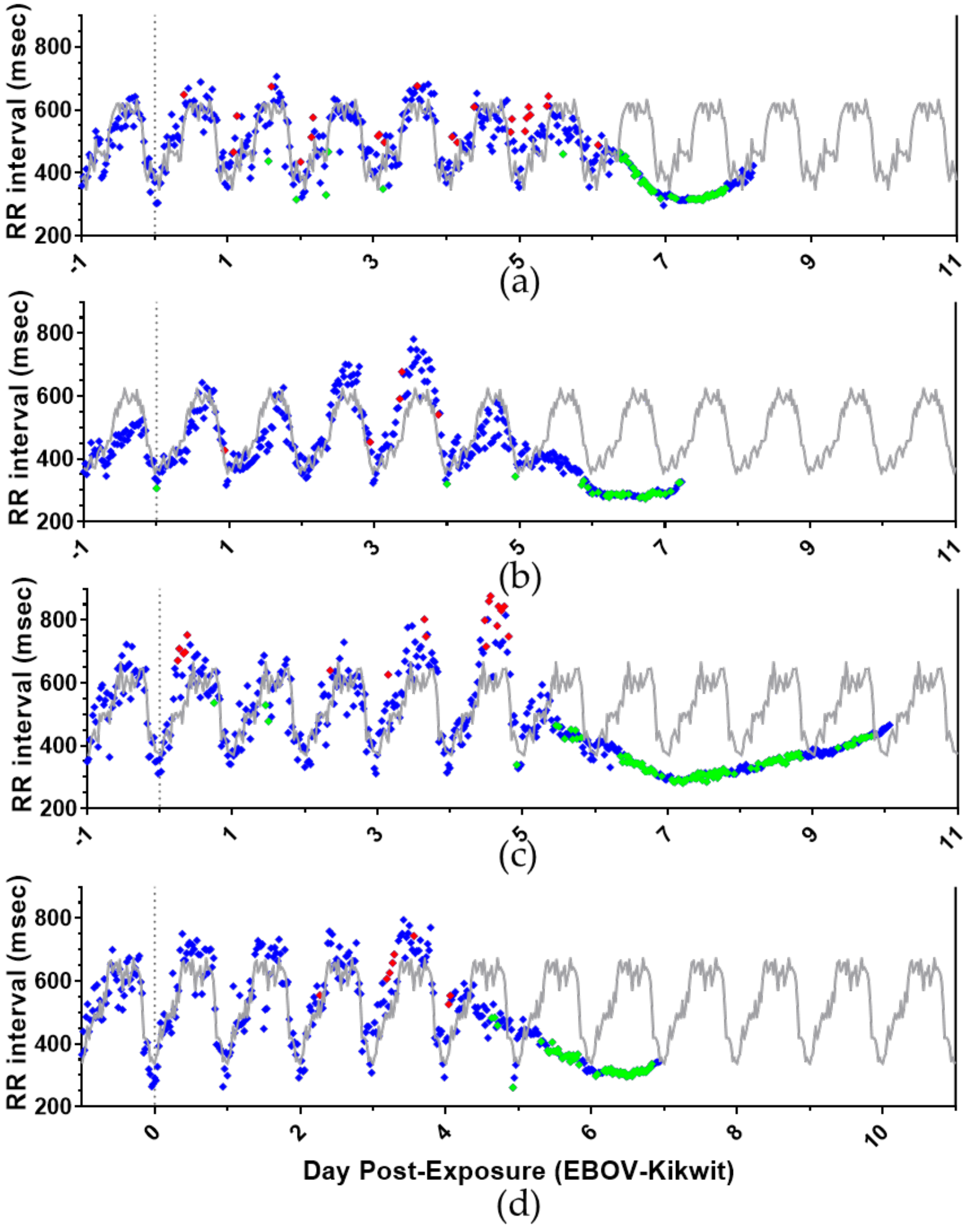

Figure S6. Telemetric RR duration detection of aerosol EBOV exposed rhesus macaques (a) NHP1 (b) NHP2 (c) NHP3 (d) NHP4. Vertical grey dotted line marks DPE 0, — BL (Baseline), ♦ HS (Values significantly higher: >3.0 SD from corresponding baseline), ♦ LS (Values significantly lower: < 3.0 SD from corresponding baseline), ♦ (Values within 3.0 SD from corresponding baseline) for RR duration measured in milliseconds.

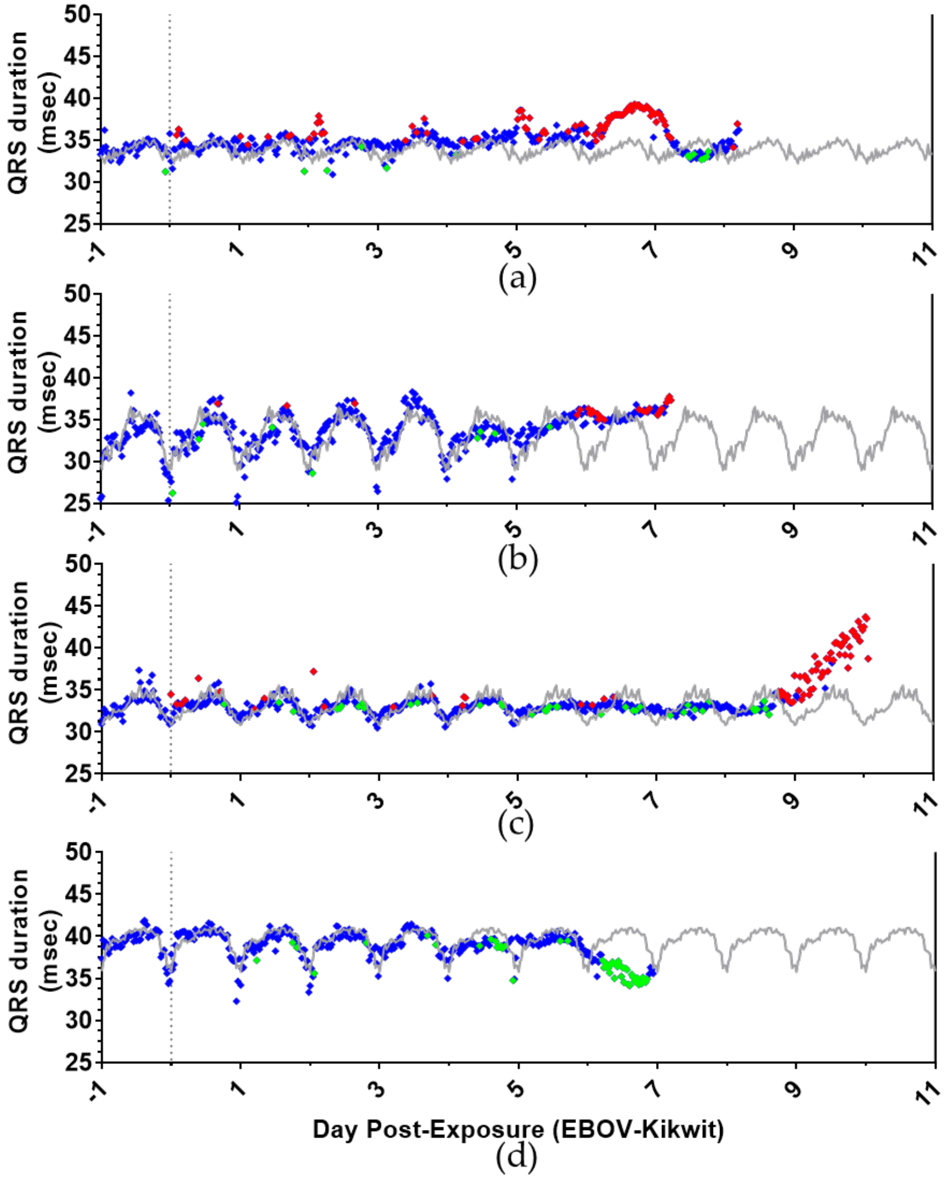

Figure S7. Telemetric QRS duration detection of aerosol EBOV exposed rhesus macaques (a) NHP1 (b) NHP2 (c) NHP3 (d) NHP4. Vertical grey dotted line marks DPE 0, — BL (Baseline), ♦ HS (Values significantly higher: >3.0 SD from corresponding baseline), ♦ LS (Values significantly lower: < 3.0 SD from corresponding baseline), ♦ (Values within 3.0 SD from corresponding baseline) for QRS duration measured in milliseconds.

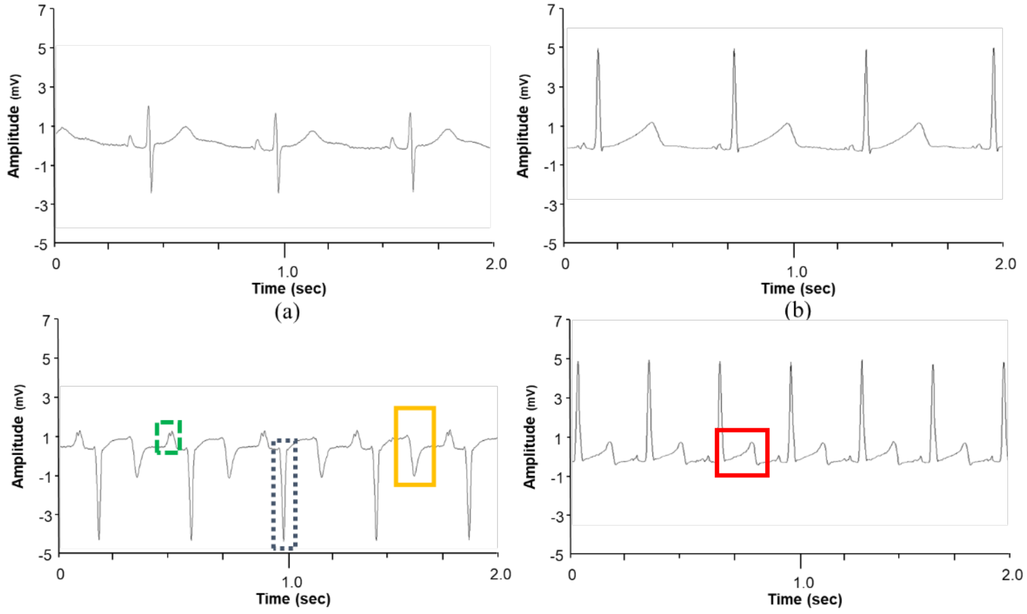

Figure S8. ECG highlights. (a) NHP1 Baseline, prior to challenge. (b) NHP2 Baseline, prior to challenge. (c) NHP1 DPE 8 with P wave morphology changes (dash green line), QRS complex morphology changes (dot grey line), and T wave inversions (solid orange line). (d) NHP2 DPE 7 showing ST segment morphology changes (solid red line) compared to baseline.

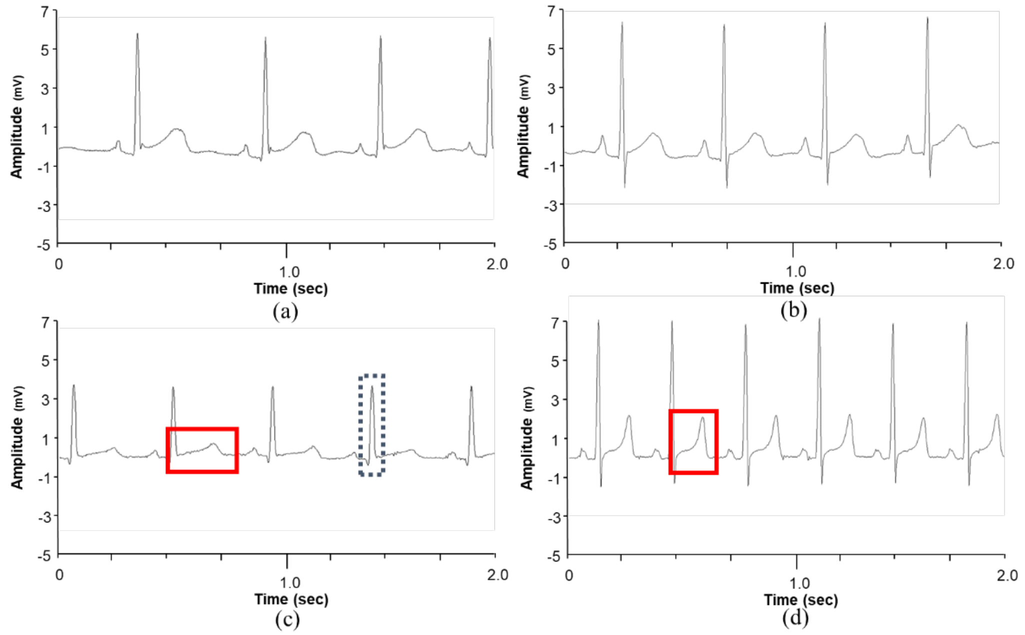

Figure S9. ECG highlights. (a) NHP3 Baseline, prior to challenge. (b) NHP4 Baseline, prior to challenge. (c) NHP3 DPE 10 with ST segment morphology change (solid red line), decreased QRS amplitude (dot grey line). (d) NHP4 DPE 7 showing ST segment morphology changes (solid red line) compared to baseline.

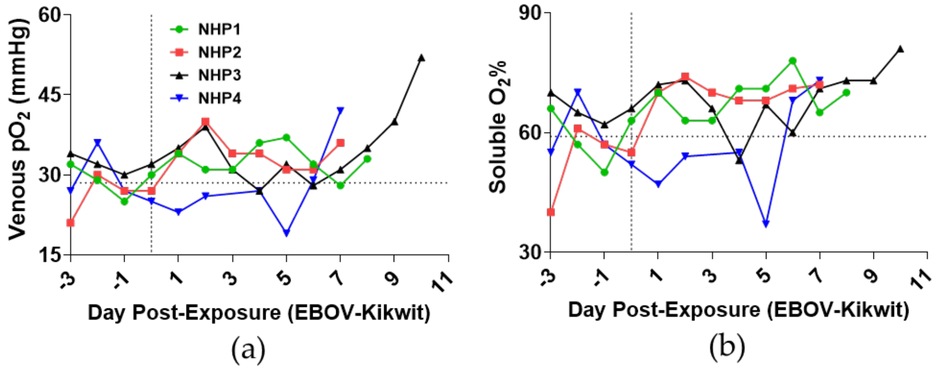

Figure S10. Time course of venous partial pressure and soluble percentage of oxygen in EBOV-challenged rhesus macaques. Detection of (a) partial pressure of oxygen (pO_2) and (b) soluble oxygen percentage (sO_2%) in whole blood using i-Stat CG4+. DPE 0 is marked by a black vertical dot line. The black horizontal dashed line is the average of all animals for DPE 0. (●NHP1) (■NHP2) (▲NHP3) (▼NHP4)

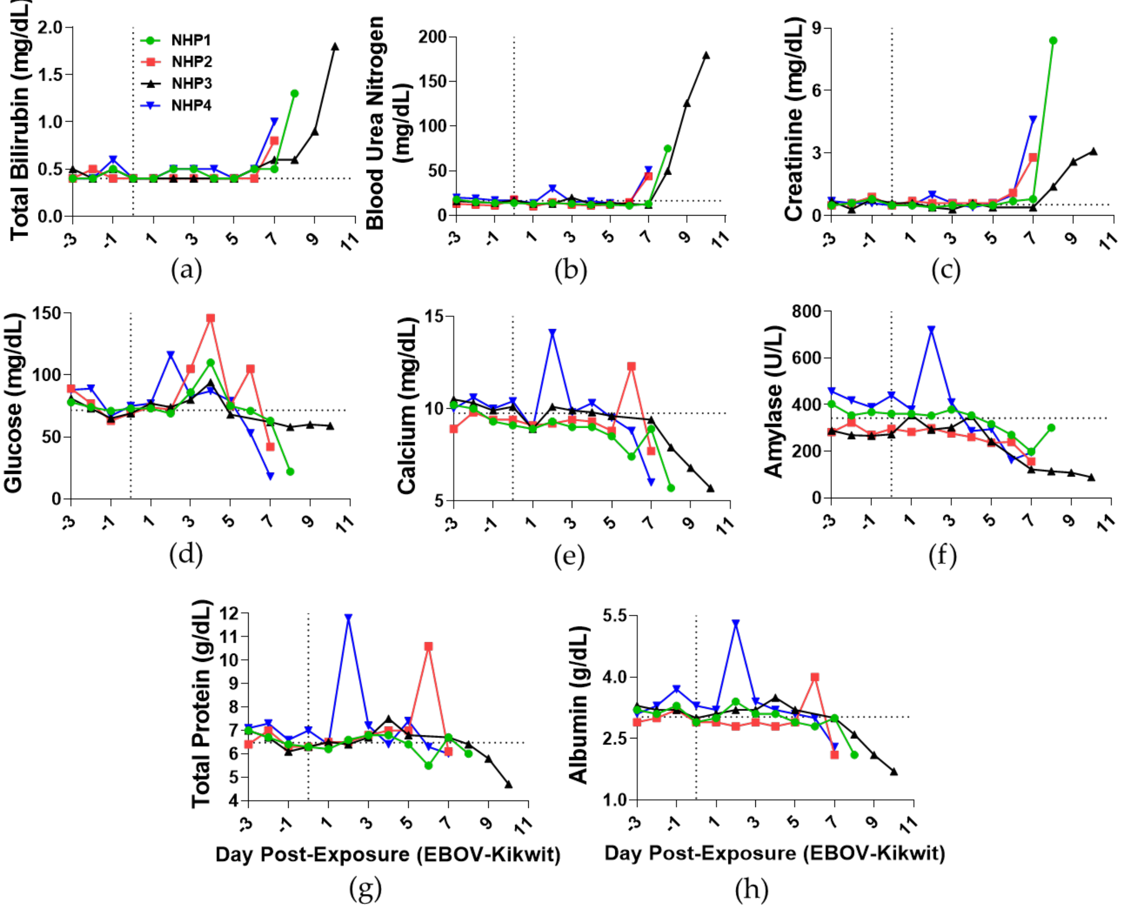

Figure S11. Time course of clinical chemistry of EBOV challenged rhesus macaques. Serum (a) total bilirubin (b) blood-urea–nitrogen, (c) creatinine, (d) glucose, (e) calcium, (f) amylase, (g) total protein, and (h) albumin levels determined by Piccolo analysis. DPE 0 is marked by a black vertical dot line. The black horizontal dashed line is the average of all animals for DPE 0. (●NHP1) (■NHP2) (▲NHP3) (▼NHP4).

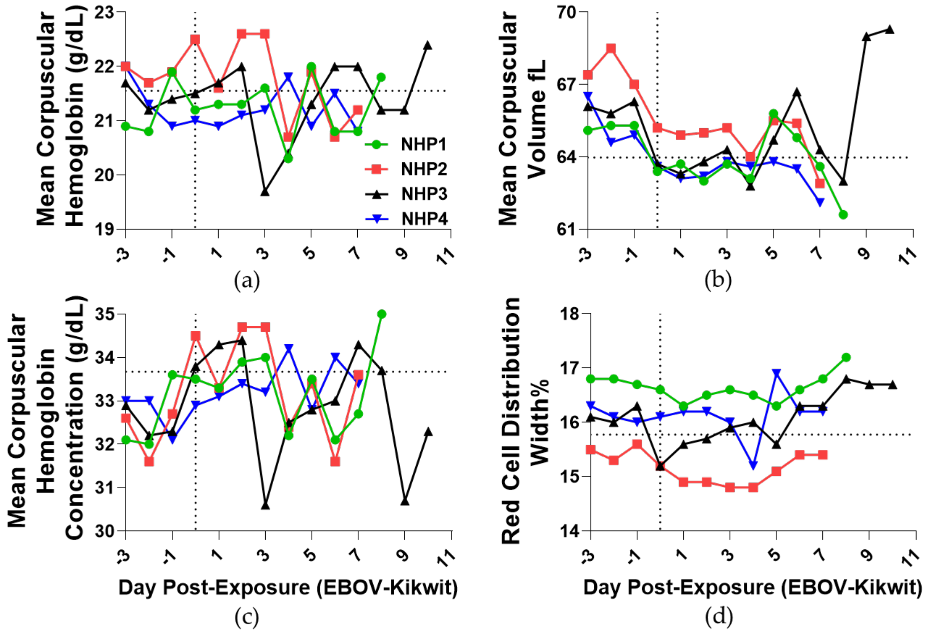

Figure S12. Time course of hematology of EBOV challenged rhesus macaques. Serum (a) mean corpuscular hemoglobin, (b) mean corpuscular volume, (c) mean corpuscular hemoglobin concentration, (d) red cell distribution width %, determined by Hemavet analysis. DPE 0 is marked by a black vertical dot line. The black horizontal dashed line is the average of all animals for DPE 0. (●NHP1) (■NHP2) (▲NHP3) (▼NHP4).

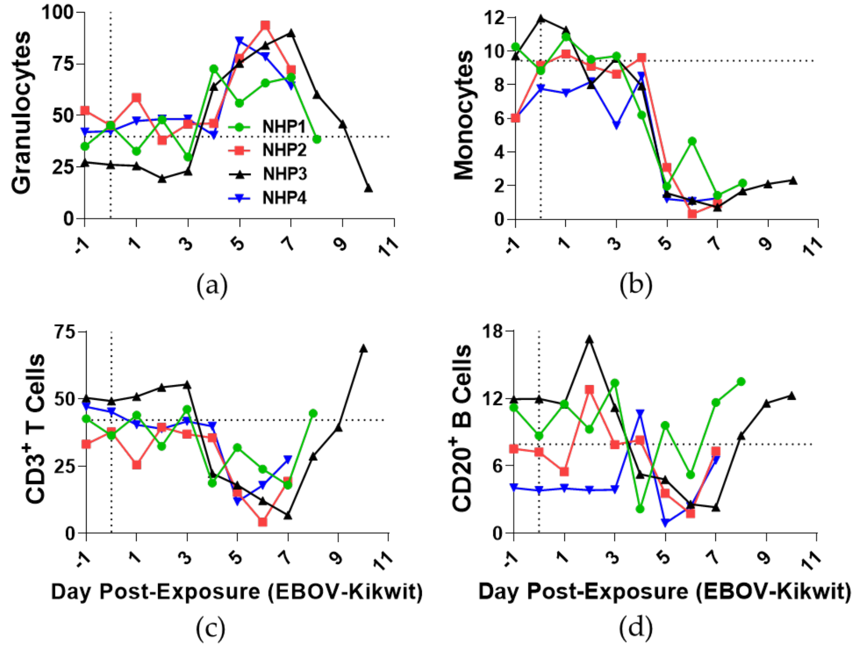

Figure S13. Flow cytometric analysis of white blood cells of aerosol EBOV-challenged rhesus macaques. Percentage of blood (a) granulocytes, (b) monocytes, (c) $CD3^+$ T cells, and (d) $CD20^+$ B cells from day DPE -1 through DPE 10. DPE 0 is marked by a black vertical dot line. The gray horizontal dashed line is the average of all animals for DPE 0. (●NHP1) (■NHP2) (▲NHP3) (▼NHP4).

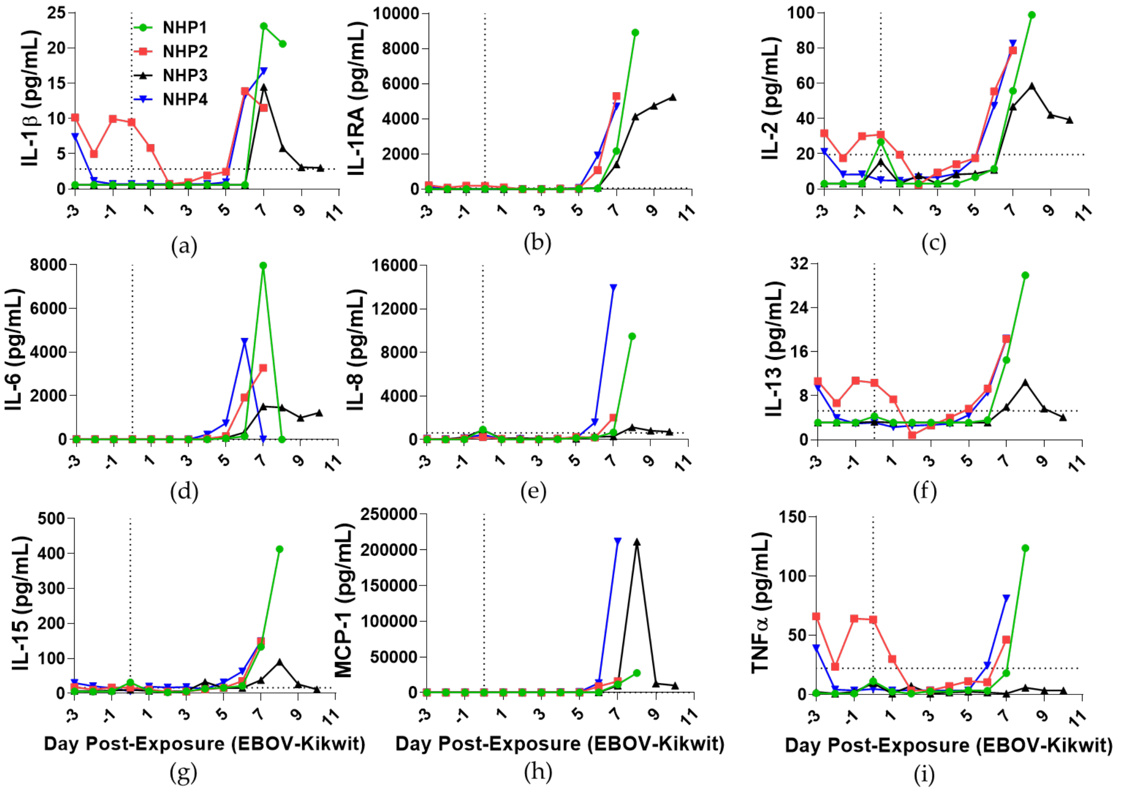

Figure S14. Assessment of blood cytokines levels of aerosol EBOV-challenged rhesus macaques. Analysis was performed on samples for (a) IL-1β, (b) IL-1RA, (c) IL-2, (d) IL-6, (e) IL-8, (f) IL-13, (g) IL-15, (i) MCP-1, and (j) TNFα, collected on DPE -3 through DPE 10. DPE 0 is marked by a black vertical dot line. The black horizontal dashed line is the average of all animals for DPE 0. (●NHP1) (■NHP2) (▲NHP3) (▼NHP4).

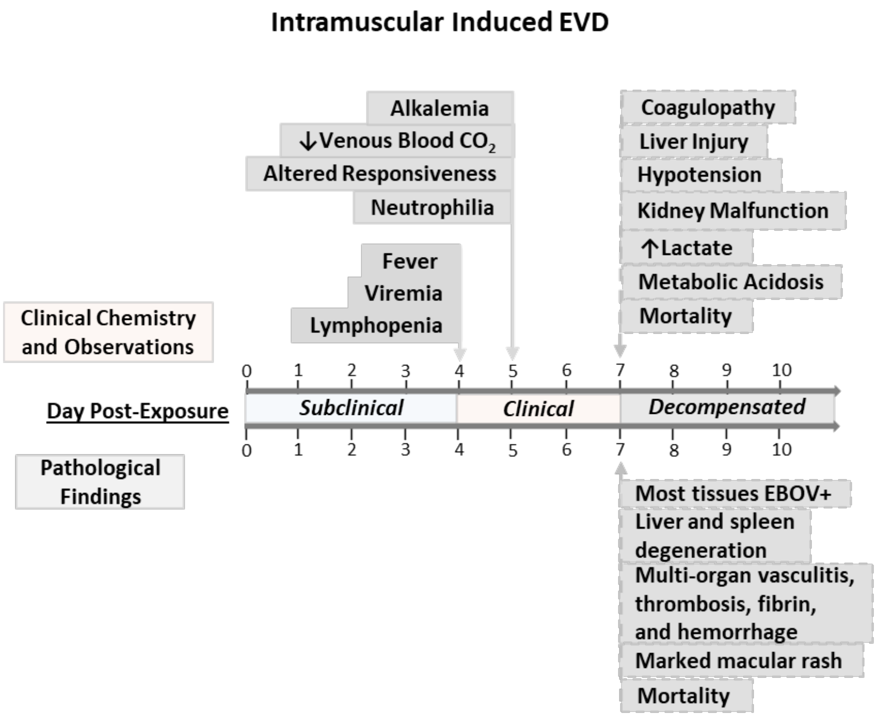

Figure S15. Comparison of natural history of intramuscular induced Ebola Virus Disease in rhesus macaques. Historic intramuscular EBOV natural history studies of rhesus macaques from Warren *et al*. 2020 and Kortepeter *et al*. 2011 have shown no distinct clinical signs between DPE 0-3 [23, 37]. In accordance with parameters in the current study, the clinical stage of disease progression is defined by fever initially occurring on DPE 4. This fever was accompanied by viremia and lymphopenia. Neutrophilia, altered responsiveness, tachypnea, low carbon dioxide levels, and alkalemia (pH>7.5) were observed on DPE 5. The decompensated stage is identified by coagulopathy, liver injury, and kidney malfunction. It is followed by hypotension, elevate lactate concentrations, metabolic acidosis (pH<7.4), and ultimately death between DPE 7-10. Pathological findings of intramuscular serial sacrifice studies were not found within the required study parameters. Based on aerosol target dose of 100 PFU, the characteristics of aerosol and intramuscular induced EVD in NHPs are reported nearly identical when respectively comparing peak viremia, infectious virus profile, survival, and time to death [50]. (solid gray arrow = start of clinical stage) (solid gray line = clinical stage) (dashed gray arrow = start of decompensated stage)
